# High-yield spidroin mimics for bioinspired fibers via computational design

**DOI:** 10.3389/fbioe.2025.1587546

**Published:** 2025-04-24

**Authors:** Yufan Huang, Junzi Qi, Bingrui An, Bensheng Zhang, Yukang Yang, Cheng Cheng, Bingfang He

**Affiliations:** ^1^ College of Biotechnology and Pharmaceutical Engineering, Nanjing Tech University, Nanjing, China; ^2^ School of Pharmaceutical Sciences, Nanjing Tech University, Nanjing, China

**Keywords:** spidroin, β-sheet, coarse-grained molecular dynamics, *Escherichia coli*, heterologous expression, self-assembly

## Abstract

The exceptional mechanical properties, biocompatibility, and biodegradability of spider silk make it a promising biomaterial, yet large-scale production remains hindered by challenges in heterologous expression. Existing prokaryotic systems face bottlenecks due to spidroins’ high molecular weight, repetitive sequences, and GC-rich motifs, leading to low yields, premature transcription termination, and insoluble inclusion bodies. Addressing these challenges, the study integrates deep learning and bioengineering to design water-soluble, β-sheet-rich spidroin mimics optimized for efficient prokaryotic expression. By replacing polyalanine motifs in *Nephila clavipe*s MaSp1 with computationally screened sequences (e.g., ITVQQ from *Burkholderia* OspA), five functional spidroins were engineered and solubly expressed in *E. coli*, achieving yields up to 0.99 g/L. Circular dichroism revealed that modified spidroins (e.g., 3rep-ITVQQ) exhibited β-sheet content up to 81.3% under thermal induction, surpassing unmodified MaSp1 (41.5%). Structural analysis via SEM demonstrated dense, uniform networks in 3rep-ITVQQ, correlating with enhanced mechanical potential. And 24rep-ITVQQ nanofibers were successfully prepared by electrostatic spinning. Coarse-grained molecular dynamics simulations validated progressive self-assembly with reduced solvent-accessible surface area over 1,000 ns. This work bridges the gap between sequence design and scalable production by overcoming expression barriers, simplifying purification, and leveraging β-sheet stacking for tunable mechanical properties. The results provide a blueprint for high-performance biomimetic fibers, advancing applications (e.g., surgical sutures, scaffolds) in tissue engineering and functional materials while addressing the limitations of conventional spidroin production systems.

## Introduction

As one of the protein fibers with the most superior mechanical properties, spider silk has excellent biocompatibility, biodegradability, and hypoallergenicity. Therefore, it has received widespread attention and applications in various fields, such as biomedicine and tissue engineering (De Jaeghere et al., 2013; Keten et al., 2010). There are seven secretory glands in spiders that secrete seven different spidroins, which perform various functions in the survival of spiders (Jaleel et al., 2020). Among them, Major ampullate silk, also known as Dragline silk (Teule et al., 2012), MaSp1, and MaSp2 constitute the main components of the Major Ampullate Spidroins (Dinjaski et al., 2017). As the framing silk of spider lifelines and webs, it is much stronger than nylon and steel and is biodegradable and compatible (Giesa et al., 2016; Heim et al., 2009), showing great potential in biomaterials (Marhabaie et al., 2014). However, as a predatory animal, *N. clavipes* makes it difficult to meet the needs of research development and production because of its predatory habit of survival, which makes it difficult to take silk in captivity, and the heterologous expression of spidroins by using genetic engineering and biotechnology is the focus of scientific researchers’ explorations at present. In recent years, domestic and foreign researchers have successfully constructed artificial expression systems, including *E. coli*, transgenic plants, and silkworm cells, for the design and synthesis of spidroin materials (Xu et al., 2018), aiming at spinning high-performance biomimetic spider silk fibers. *E. coli* has been widely studied as a commonly used expression system, and its low production cost, ease of operation, and clear genetic background make it the best choice for producing spidroin chassis cells. However, there are still multiple bottlenecks in the expression of spidroins in this system: the large molecular weight of natural spidroins and their characteristic polyalanine- and glycine-rich sequence features may lead to problems such as premature termination of transcription, inefficient expression, and the formation of inclusion body precipitates. However, through the efforts of Schmuck et al. (2021), they constructed a small molecular weight miniature spidroin (33 kDa) and achieved its high expression in *E. coli*, with a yield of up to 14.5 g/L, and prepared bionic fibers with a high toughness modulus.

In addition, it was found that the excellent mechanical properties of the main potbellied glandular filaments were attributed to the sequence structural uniqueness of its spidroins (Goldschmidt et al., 2010; Joel et al., 2023). MaSp1 mainly consists of a β-sheet crystal structure, a 3^10^-helix, and a β-turned helix. Its primary structure can be analyzed as being classified into three regions: the terminal structural domain, i.e., the C-terminal structural domain (C-Terminal, CT), and the N-terminal structural domain (N-Terminal (N-Terminal, NT) and the repetitive core domain (Rep). The two non-repeat structural domains play a crucial role in the assembly of silk fibers in response to the environment (Heim et al., 2009; Rising and Johansson, 2015; Vollrath and Knight, 2001; Yarger et al., 2018). In contrast, it is worth mentioning that the core structural domain dominates the amino acid composition of spidroins, accounting for about 90%, which constitutes the main body of the spidroins sequence and plays a decisive role in the physical properties of silk fibers (Simmons et al., 1994).

The repeat region consists of alternating microcrystalline and non-microcrystalline regions (usually present in amorphous aggregates) and contains regular characteristic motifs A_n_ (n:6–14), (GA)_n_, and GGX (Hayashi et al., 1999). Among them, GGX in the non-microcrystalline region mainly forms a 3^10^-helix structure, which determines the high ductility and high Young’s modulus of the spider silk (Han et al., 2013; Tokareva et al., 2014), whereas A_n_ and (GA)_n_ in the microcrystalline region can form a reverse-parallel β-sheets, and the peptide chains are linked via hydrogen bonds, forming dense lamellar structure with strong intermolecular forces, which endows the spider silk with high strength and high modulus (Lefevre et al., 2011; Roemer and Scheibel, 2008). The eukaryotic expression system fundamentally differs from prokaryotic systems in protein synthesis mechanisms. In spider silk glands, ribosomes responsible for spidroin synthesis are anchored to the endoplasmic reticulum (ER), requiring nascent silk proteins to traverse the ER membrane into the glandular lumen. Alanine (Ala), possessing moderate β-sheet-forming capacity with efficient ER membrane permeability, evolutionarily developed highly polymerized polyalanine(A) n repeats. In contrast, hydrophobic residues like valine (Val) and isoleucine (Ile) exhibiting stronger β-sheet propensity tend to accumulate within hydrophobic membrane domains. Conversely, prokaryotic systems (e.g., *E. coli*) conduct cytoplasmic protein synthesis liberated from poly(A) n constraints, enabling a broader selection of amino acids with enhanced β-sheet propensity. Leveraging these metabolic and biosynthetic features of *E. coli*, we implemented microcrystalline domain engineering to achieve high-yield production of biomimetic spidroins.

$$\left[{\left(A\right)}_{n}/{\left(GA\right)}_{n...}\right]\left[{\left(GGX\right)}_{n}/{\left(GPGXX\right)}_{n}/{\left(GPGQQ\right)}_{n...}\right]$$

(Heim et al., 2009)

Before the formation of silk fibers, spidroins are stored in the spider glands as highly concentrated solutions (30%–50%). During spinning, the soluble spidroins undergo conformational changes and molecular rearrangements. When subjected to external stimuli such as acidification and shear forces (Andersson et al., 2014; Knight and Vollrath, 2001; Tillinghast et al., 1984; Sparkes and Holland, 2017), spidroins change from structures such as irregularly curled and α-helices to dense β-sheet structures, and their tight arrangement confers the properties of the spring structure of the molecules in the semi-crystalline state of spidroins. Therefore, the structural stability of β-sheet directly affects the mechanical properties of spider silk (Mohammadi et al., 2019). Liu et al. illustrated the structure and formation process of β-crystals and nano-fishing webs and deeply analyzed the effect on mechanical properties, which provides a new strategy for the design and synthesis of high-performance fibers (Liu et al., 2016). In the formation process of β-sheet, amino acid residues in proteins are connected through high-density hydrogen bonding, forming β-crystals that resemble layers stacked on top of each other. With their continuous growth and cross-linking, they eventually form the fishing net structure in spider silk nanofiber bundles. This structure not only maintains the elasticity of the spider silk fibers but also effectively disperses the effect of external stress on the entire structural network, further enhancing the mechanical properties of the silk fibers (Lefevre et al., 2011). The highly ordered β-sheets structure confers excellent mechanical properties to natural materials (Ge et al., 2020). Among them, the content of β-sheet is particularly important. In Chan et al.'s study, by introducing β-sheet nanocrystals into the soft polymer network, the hardness of the prepared hydrogel would be enhanced with the increase of the number of β-sheets, so it increased its compressive and compressible capacity. These properties can be adjusted by adjusting the content of β-sheets (Chan et al., 2020). A large number of β-sheets can gradually stack and form strong crystals, and researchers have taken advantage of this property by replacing the layered structure of natural heart valves with silk proteins containing stacked β-sheets, which showed an increase in stacking strength of 292%. Compared with the natural silk protein, the strength was increased by 1,380%, and the performance of the valve they prepared can replace human heart valves (Choi et al., 2024).

Amyloid has a β-sheet structure, which is its typical structural feature. It is arranged in the form of β-sheet layers that interact to form oligomers, which later further polymerize to form amyloid fibrils. These fibers exhibit interlaced β-sheet segments resembling a helical structure (Purro et al., 2018). Li et al. utilized amyloid peptide motifs that can form β-sheets linked to spidroins’ glycine-enriched flexible motifs to confer mechanical strength on a macroscopic scale (Li et al., 2021). The fibers they prepared surpassed most recombinant protein fibers and even some natural arachnid fibers in terms of tensile strength and toughness. However, amyloid is expressed in inclusion bodies and requires washing and purification using organic solvents, which inevitably poses a risk of toxicity to humans.

Currently, our knowledge of cross-β-sheets protein sequences is still lacking, but obtaining cross-β-sheet motifs with good characterization properties and excellent water solubility is the basis for our further knowledge of the relationship between structure and performance (Biancalana et al., 2010). This is an important reason why we chose sequences from these sources. Functional and soluble arachnid analogs are expected to accelerate the relevant applications of spidroins, but effective and convenient design strategies are lacking. In this study, sequences with high potential to form β-sheets were collected based on a deep learning strategy (Biancalana et al., 2010). The selected sequences were mainly derived from the Outer Surface Protein A (OspA), one of the major outer membrane proteins of *Burkholderia sparsalis*, which has unique structural and functional properties. OspA consists of 21 consecutive reverse-parallel β-sheets and one α-helix located at the C-terminal end, and its β-sheet plays an important role in the maintenance of protein stability and function. The content and type of β-sheets in the microcrystalline regions of spidroins are the key factors determining the self-assembly performance and mechanical strength of spider silk. Of this property, we designed five water-soluble and functional mimetic spidroins and characterized their solubility and, self-assembly and membrane applications. This study increases the diversity of backbone sequences as well as ensures their easy expression and solubility and provides a reference for the design of recombinant spidroins, mussel proteins, collagens, and other proteins with highly repetitive sequences.

## Materials and methods

### Materials and reagents

Plasmid pSE380 was purchased from Vazyne, Inc. One-step cloning kit, restriction endonuclease, and ligase was purchased from Takara, and sequencing was done by Nanjing General Biotechnology Co. Chemoreceptor *E. coli* BL21 (DE 3) cells were purchased from Vazyne, Inc. The repeat region (Rep) comes from the *Euprosthenops australis* MaSp1 sequence. It has been converted from two polyalanine regions to three polyalanine regions according to the experience design (EMBL accession number AJ973155). Replace the polyalanine region therein with the screened fragments (GVLEGV, KTAAWN, ITVQQ). NT3repCT was synthesized by GenScript (Nanjing). Company, and NT was derived from *E. australis* MaSp1 sequence (GenBank accession number AM259067). CT is derived from the *Araneus ventricosus* MiSp sequence (GenBank accession number JX513956). To obtain the 6rep sequence, NT6repCT was generated by duplicating the intermediate domain of recombinant spidroin using NheI and SpeI restriction sites and a digestion-ligation strategy. Cell lysis was performed in an ultrasonic cell breaker (Ningbo Xinzhi Bio-technology Co., Ltd.). Protein purification on all Ni-NTA columns was performed on an AKTA protein purifier. Freeze-drying of the purified proteins was performed on a freeze-dryer (Ningbo Xinzhi Bio-technology Co., Ltd.).

### Preparation of artificial spider silk solution

Single colonies of *E. coli* BL21 (DE3) harboring chimeric spidroin constructs (3rep-GVLEGV, 3rep-KTAAWN, 3rep-ITVQQ) were inoculated into LB medium (10 g/L tryptone, 5 g/L yeast extract, 10 g/L NaCl) and incubated overnight at 37°C with 180 rmp shaking. The culture was then subcultured into fresh LB medium, and protein expression was induced by adding 0.1–0.5 mM IPTG when the OD_600_ = 0.6–0.8. For systematic process optimization, a full factorial design encompassing IPTG concentration (0.1–1.0 mM), induction temperature (20°C–35°C), and duration (6–36 h) was implemented. Post-induction cultures were harvested by centrifugation (8,000 rpm, 10 min, 4°C) after 24 h at 30°C baseline conditions, with cell pellets stored at −80°C for subsequent analysis.

### Protein purification

Recombinant spidroins were purified by nickel column affinity chromatography. First, the cells were resuspended in pure water, followed by cell fragmentation and centrifugation. The Ni-NTA column was loaded with supernatant, and the column was washed with B (500 mM/L imidazole, 20 mM Tris, 300 mM NaCl, pH 8.0) buffer, and the eluate was collected. Protein elution was performed using a stepwise imidazole gradient. The column was sequentially washed with binding buffer containing 25 mM (5%), 50 mM (10%), 75 mM (15%), 150 mM (30%), 300 mM (60%), and 500 mM (100%) imidazole. All proteins can be eluted off at 150 mM or 300 mM concentrations. 24rep-ITVQQ was expressed in inclusion bodies, with the purification procedure following the protocol as described elsewhere (Cai et al., 2020). All SDS-PAGE gels were 1 mm thick discontinuous gels with a 4% stacking gel at the top and a 12.5% separation gel at the bottom. Protein samples were prepared in 4× protein sample buffer (60 mm Tris pH 6.8, 10% glycerol, 2% SDS, 0.01% bromophenol blue, 100 μm DTT). Gel electrophoresis on polyacrylamide gels was performed in 1 × Tris-glycine SDS buffer (25 mM Tris, 250 mM glycine, 0.1% w/v SDS) in gel electrophoresis apparatus (Bio-Rad). The protein expression level was estimated by counting the intensity of the product bands as the sum of the intensities of all protein bands on the gel. Purified recombinant proteins were obtained by overnight dialysis in purified water at 4°C.

### SEM analysis

Use the Hitachi S-4800 Field Emission Scanning Electron Microscope to observe the surface morphology and structure of samples. The device employs a cold field emission electron source with excellent high resolution and advanced detection technology. Its secondary electron image resolution is up to 1.0 nm at high accelerating voltage (15 kV) and 1.4 nm at low accelerating voltage (1 kV) This high resolution enables the S-4800 to capture the microscopic details of samples, making it suitable for observing nanomaterials and biological samples, among others.

### Circular dichroism analysis

A Jasco J-1500 spectrometer was used to carry out circular dichroism (CD) spectra at 25°C–85°C. The self-assembled spidroins were diluted to 0.1 mg/mL for measurement using glass cuvettes with a path length of 1 cm. Wavelength scans were collected from 185 to 260 nm in 0.5 nm steps with a scan speed of 100 nm/min and a bandwidth of 1.0 nm. The CD spectral data were smoothed and pre-processed using spectra manager software, and the processed data were imported into CDPro software, where the CONTIN method was selected, the wave number range was chosen from 190 to 240 nm, and the fitting calculation was carried out to obtain the relative content of the secondary structure.

### Self-assembly capability analysis

To evaluate the self-assembly properties of the recombinant spidroin 24rep-ITVQQ, its fine morphology was analyzed using Atomic Force Microscopy (AFM). The samples were examined with a Bruker ICON-type atomic force microscope. Initially, the protein stock solution was diluted to a final concentration of 0.001% (w/v). Subsequently, 20 μL of this solution was uniformly applied dropwise onto the surface of a mica sheet and allowed to dry at room temperature before testing. For the analysis, scanning mode was selected, and a probe with a curvature radius of 2 nm (elasticity coefficient of 0.4 N/m) was utilized. The surface morphology and mechanical property data of the nanofibers were reconstructed in three dimensions and quantitatively analyzed using NanoScope Analysis version 1.8 software.

### Fabrication of 24rep-ITVQQ electrospun nanofibers

A solution was prepared by dissolving 200 mg of solid 24rep-ITVQQ in 2 mL of 1,1,1,3,3,3-hexafluoro-2-propanol (HFIP, Macklin). The resulting solution was then injected through a capillary tip using a 10-mL syringe. During the electrospinning process, a high voltage of 17 kV was applied to the needle, and the flow rate of the spinning solution was maintained at 2 mL/h. The electrospun nanofibers were collected on aluminum foil paper and the electrospun fibers were freeze-dried, followed by observation of the nanomorphology of the fibers using SEM.

### Molecular dynamics simulation

The structure of 24rep-ITVQQ was obtained by Alphafold3. Molecular dynamics simulations were performed using Gromacs2021 software, and the force field used for the system was the Martini force field. The molecules of the system were placed in the box using the Gromacs construction box, and then the coarse-grained system was constructed using the MARTINI Maker in CHARMM-GUI. Simulations were performed at 298.15 K temperature and 1 bar pressure. Energy minimization, NVT pre-equilibration, and NPT pre-equilibration were performed sequentially before the simulation, and the finished simulation was performed for 1000 ns after the system was relaxed. The finished simulation integrates Newton’s equations of motion using the frog hopping algorithm with a time step of 20 fs. The finished simulation uses the V-rescale temperature coupling method and the Parrinello-Rahman pressure coupling method. The nearest neighbor search is performed using the Verlet method with a Coulomb and van der Waals interaction truncation radius of 1.1 nm.

## Results

### Design and construction of biomimetic spidroins

In this study, we first collected information on β-sheet feature motifs from different biological sources from the NCBI/PDB database. Then, the computer in house algorithm was used for intelligent screening to extract and classify the data, and the β-sheet feature sequence database “o” is established. To verify the feasibility of β-sheet in the screened sequences, we selected five groups of β-sheet feature motifs, the selected soluble recombinant spidroin motifs are shown in Table 1. Reasons for screening are as follows:

(1) Motif GVLEGV: This motif appears 26 times in the selected database, exhibiting a significantly high frequency. This suggests that its amino acid composition and spatial arrangement have a strong tendency to form β-sheet structures, potentially achieving secondary structure stability through stable hydrophobic core formation and hydrogen bond network optimization.
(2) Motif ITVQQ: This motif is characterized by low abundance (<5%) in microcrystalline regions due to the glutamine diplet (QQ). By introducing charge modifications or polar-regulated residue substitutions, we evaluated the structural adaptability of the sequence in the amorphous phase environment. The aim was to elucidate the dynamic regulatory mechanism of local polarity and conformational flexibility on β-sheet formation.
(3) Motif KTAAWN: This sequence contains alanine repeat units (AA motifs) that exhibit a pronounced preference for β-sheet conformation (occurring 37 times at high frequency). Its compact side chain conformation effectively reduces steric hindrance and promotes van der Waals interactions between adjacent β-chains, consistent with the characteristics of natural spidroins.
(4) Motif SVSVSVS: The combination of valine (V) and serine (S) follows the principle of β-sheet interface complementarity. The strong hydrophobicity of V facilitates core layer formation, while the hydroxyl group of S enhances interchain binding energy through hydrogen bonding.
(5) Motif VVVKI: This sequence has a high valine content (68.4%). As a hydrophobic amino acid, valine plays a crucial role in β-sheet formation, enhancing structural stability through increased hydrophobic interactions and side chain co-arrangement. In the absence of endoplasmic reticulum in the prokaryotic expression system of *E. coli*, the presence of β-sheet-promoting amino acids such as Val and Ile may lead to the formation of more robust super-β-sheet crystals.

**TABLE 1:** Screening of soluble β-sheet motifs of recombinant spidroin.

Source	PDB ID	β-sheets motif	Function	Structure	Screening basis
*Borreliella burgdorferi*, *Borreliella burgdorferi B31*	2i5z, 3ckf, 3ckg, 2fkj, 3cka, 6j5o 6j5p, 6j5q, 5b2a, 6j5m, 6j5n, 6j6b, 6j6d, 2oy8, 2oy5, 2ol6, 2ol8	GVLEGV	Capture a self-assembly segment in a water-soluble molecule	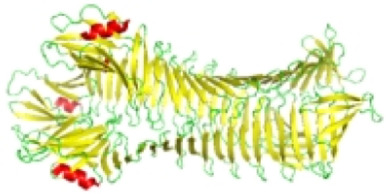	Frequency 26 times
*Borreliella burgdorferi*, *Borreliella burgdorferi B31*	2i5z, 3ckf, 3ckg, 2fkj, 3cka, 6j5o 6j5p, 6j5q, 6j5m, 2oy5, 2ol7, 2oy1, 2pi3, 2 fkg, 2hkd, 6j48, 6j5r, 5b2a 6j5n, 6j6b, 6j6c 6j6d, 6j6e, 2oy8, 2oy7, 2oyb, 6j49, 2i5v, 2ol6, 6j47 2g8c, 2af5	KTAAWN	Peptide self-assembly sequences induce structural evaluate	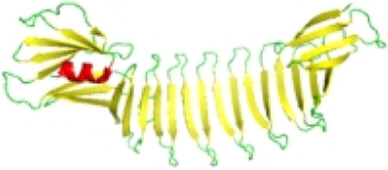	Contains AA, Frequency 37 times
*Borreliella burgdorferi*, *Borreliella burgdorferi B31*	2i5z, 3ckf, 3ckg, 2 fkg, 2fkj, 2hkd, 3cka, 6j48, 6j5o, 6j5p, 6j5q, 6j5r 5b2a, 6j5m, 6j5n, 6j6b, 6j6c, 6j6d 6j6e, 2oy8, 2oy7, 2oyb, 2oy5, 6j49, 2i5v, 2ol6, 2ol7 2ol8, 2oy1, 2pi3, 6j47, 2g8c	ITVQQ	Control nanomaterials based on β-rich peptide self-assemblies	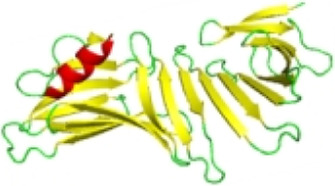	QQ occurs less frequently in the microcrystalline zone, try to see whether it can be modified
*Streptomyces globisporus*	1hzk, 1hzl	SVSVSVS	Antitumor antibiotic chromoproteins	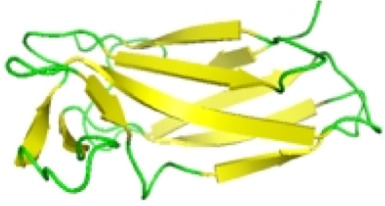	The V/S combination appeared
*Hordeum vulgare*	1ht6, 1rp9, 2qps, 1p6w, 1rp8, 3bsg, 2qpu, 3bsh, 1rpk, 3bsg	VVVKI	Inhibition of starch granule activity	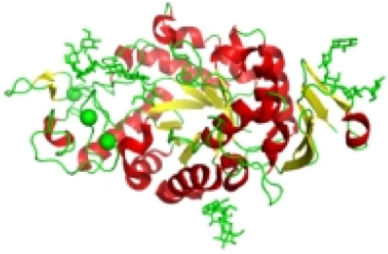	Frequency 10 times with high V content

**FIGURE 1:**
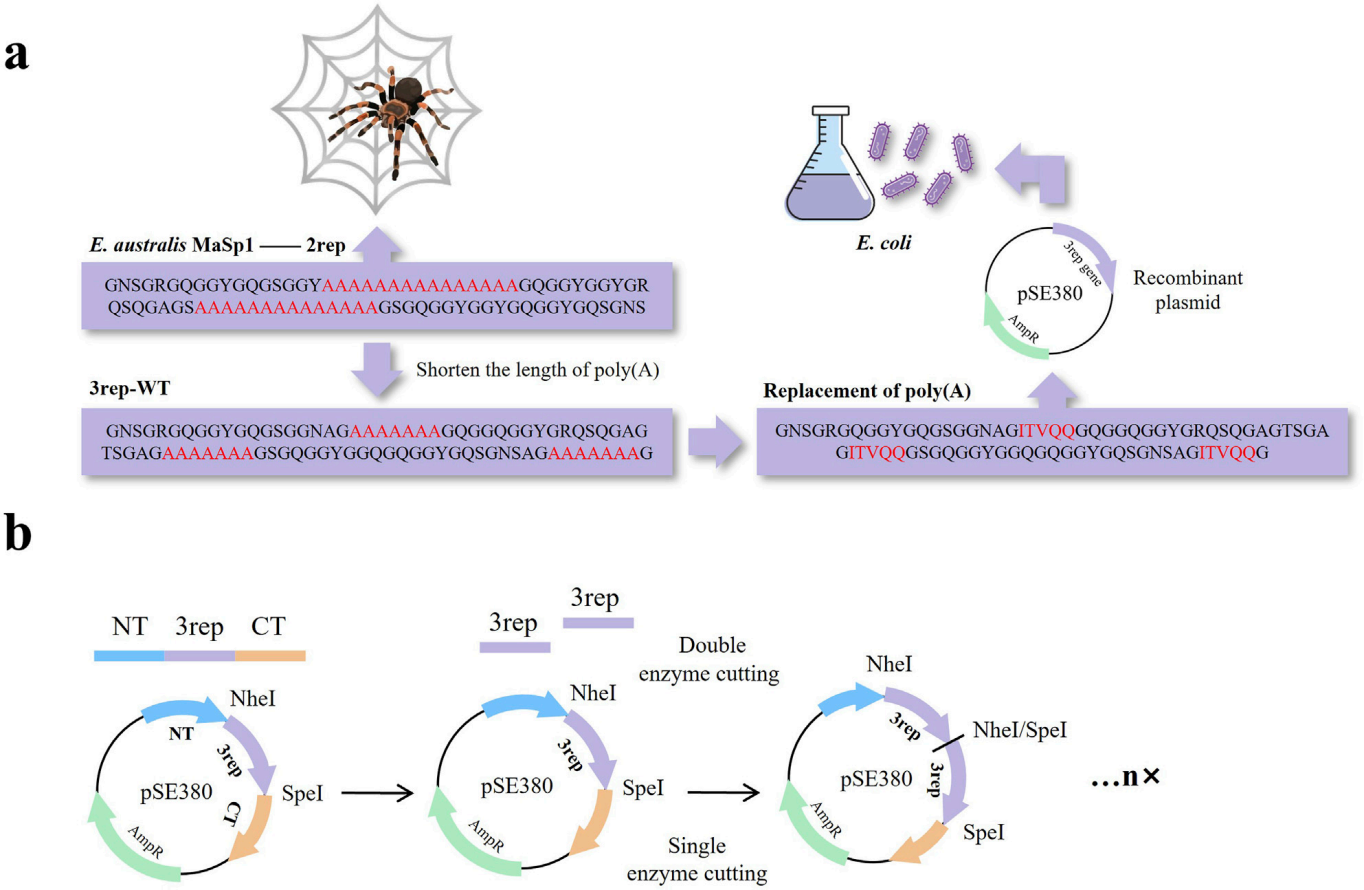
**(A)** Schematic representation of unmodified wild-type spidroins (2rep), spidroins with shortened poly(A) to reduce the difficulty of expression (3rep-WT), and recombinant mimic spidroins after replacement of poly(A) using a screened sequence (ITVQQ). **(B)** Schematic diagram of constructing multiple repeating motifs.

*Euprosthenops australis*, a group of spiders from Africa, produces MaSp1, which is known for its strong mechanical properties and is one of the most widely studied spidroins. To reduce the effect of the Poly(A) region on the expression of arachnid proteins in *E. australis*, we first selected the diploid from the repetitive structural domain of the natural spidroin *E. australis* MaSp1 as the initial template (2rep) for this study, and to make the reduction of the poly(A) repetitions, the original number of A’s of 15 was designed to be a 3rep fragment with 7 A’s (defined as 3rep-WT). Based on the 3rep-WT sequence, the polyalanine region was replaced with the selected sequence (Figure 1A; Supplementary Figure S1). Multiple repeating motifs' construction of recombinant spidroins ensures precise splicing of gene fragments and stable formation of polyploid structures by employing two restriction endonuclease sites (NheI and SpeI) that are compatible with each other but fail after a single use (Figure 1B).

### Optimization of expression conditions and purification

**FIGURE 2:**
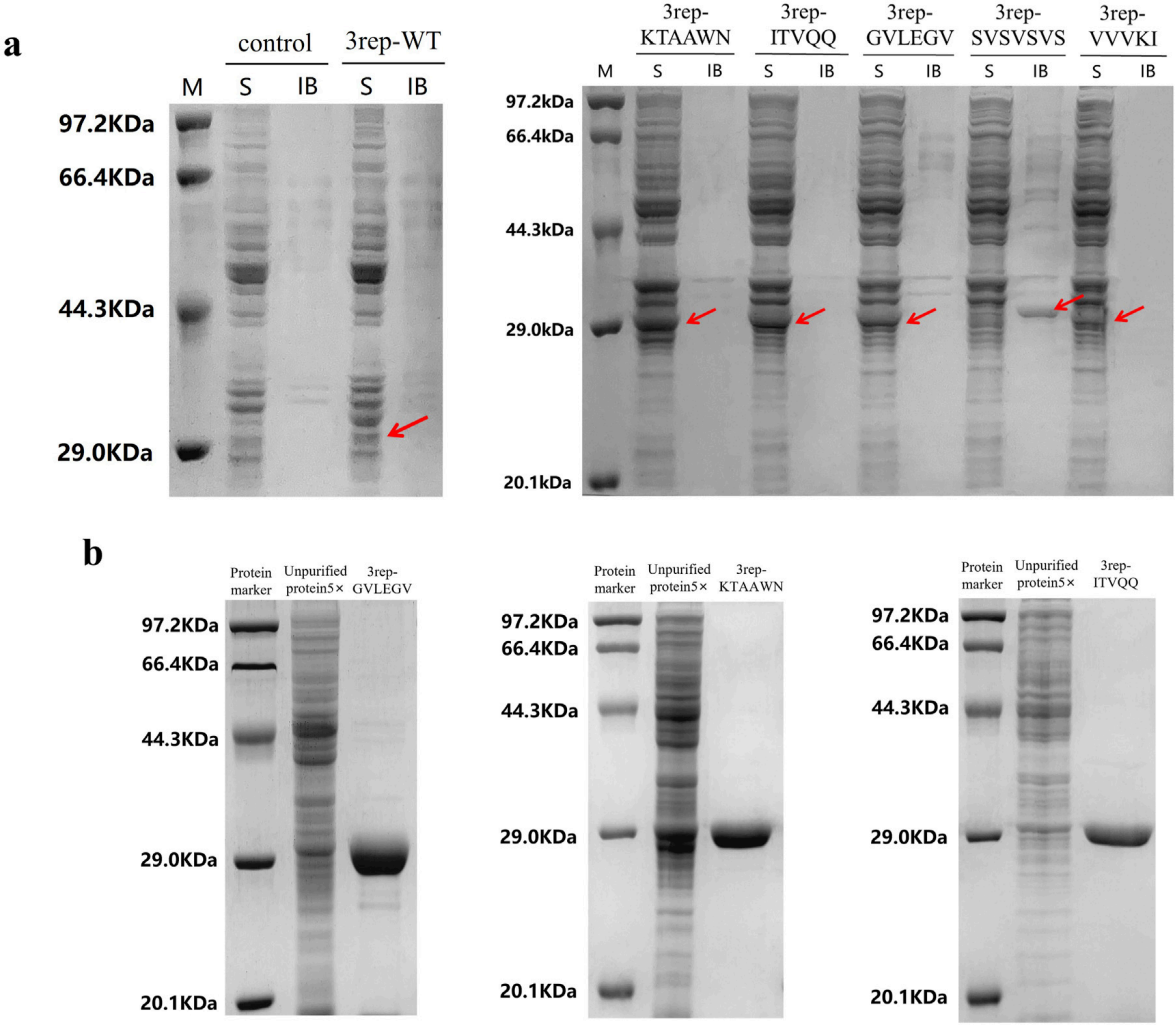
Expression and purification of recombinant spidroins. **(A)** 3rep-WT and screening of optimal protein expression conditions for recombinant spidroins 3rep-KTAAWN, 3rep-ITVQQ, 3rep-GELEGV, 3rep-SVSVSVS, and 3rep-VVVKI. **(B)** Purification of 3rep-GVLEGV (MW: 33.8 kDa), 3rep-KTAAWN (MW: 34.2 kDa), and 3rep-ITVQQ (MW: 33.9 kDa). LaneKey: protein marker (M); S stands for soluble fractions; IB stands for inclusion bodies.

Expression conditions were optimized for three chimeric spidroins, 3rep-ITVQQ at 0.1 mM IPTG induction at 30°C for 12 h, 3rep-KTAAWN at 0.5 mM IPTG induction at 30°C for 36 h, and 3rep-GVLEGV at 0.3 mM IPTG induction at 30°C for 36 h. The results are shown in Figure 2A, where the expression of recombinant spidroin 3rep-VVVKI was lower compared to the other four proteins and significantly compared to the expression of 3rep-WT. Recombinant spidroins 3rep-GVLEGV, 3rep-KTAAWN, and 3rep-ITVQQ were successfully solubilized and expressed intracellularly. Protein purification was achieved using nickel affinity chromatography, and all three recombinant spidroins could be efficiently eluted with 150 mM imidazole. Whereas recombinant spidroin 3rep-SVSVSVS existed in inclusion bodies and needed to be replicated and purified by subsequent steps. As shown in Figure 2B, grayscale analysis showed that the purity of all three types of mimic spidroins reached more than 95%. The concentrations of the three types of chimeric spidroins after purification were 480 mg/L (3rep-ITVQQ), 510 mg/L (3rep-KTAAWN), and 990 mg/L (3rep-GVLEGV), respectively. Subsequently, the three types of chimeric spidroins were dialyzed and then lyophilized overnight at 4°C for further characterization.

Lyophilized powders of 3rep-GVLEGV, 3rep-KTAAWN, and 3rep-ITVQQ were prepared by placing the fully dialyzed recombinant protein solution in freezing at 80°C overnight, followed by placing it in a freeze-dryer for 48 h and more. We used circular dichroism (CD) spectroscopy to characterize the molecular structures of these self-assembled recombinant spidrions.

**FIGURE 3:**
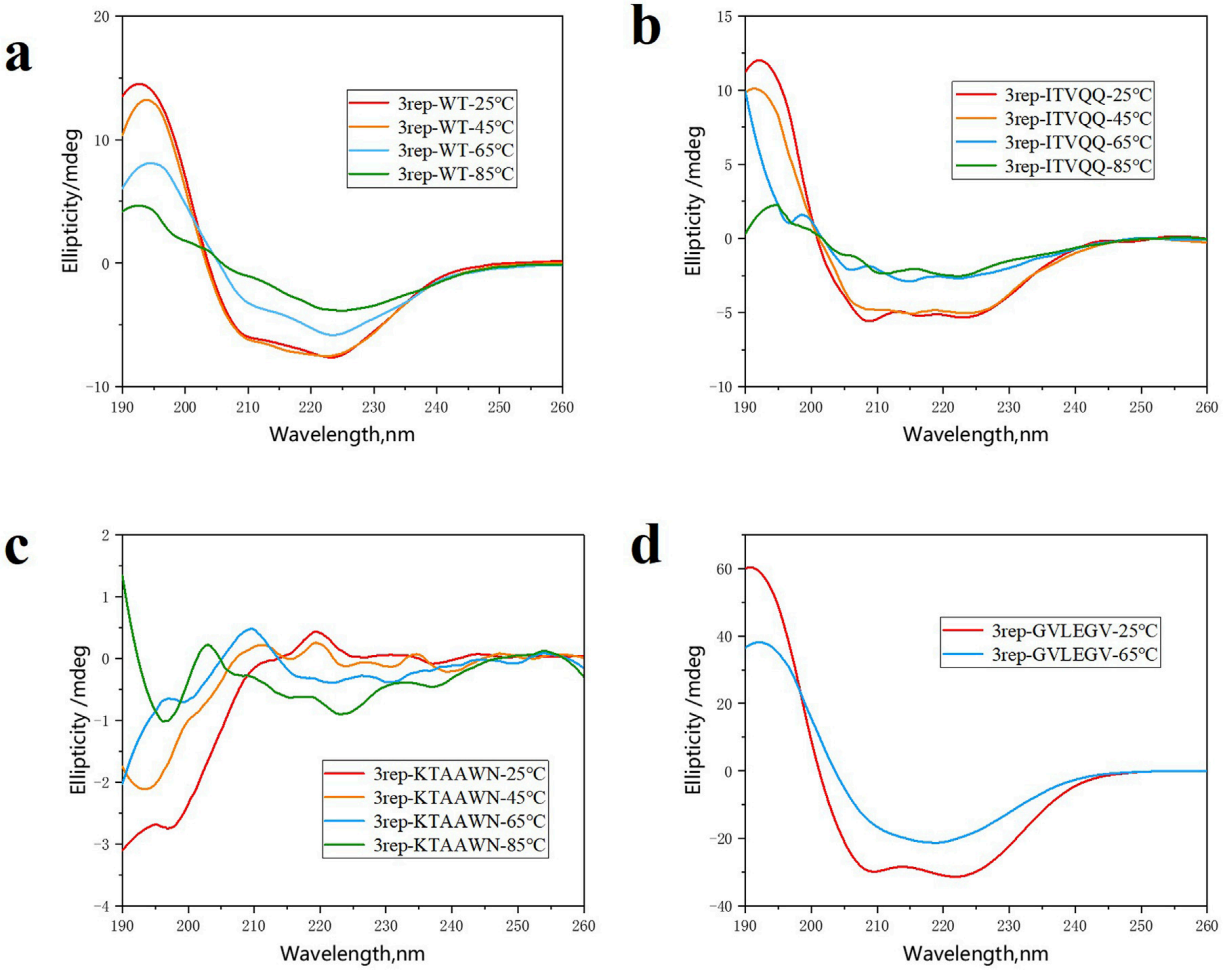
CD spectra of 3rep-WT **(A)**, 3rep-ITVQQ **(B)**, 3rep-KTAAWN **(C)**, and 3rep-GVLEGV **(D)** spidroins.

As illustrated in Figure 3, the results demonstrate that at room temperature, 3rep-WT exhibited predominantly α-helix structure, accounting for 97.7% of the total secondary structure content, with no detectable β-sheet formation. In contrast, the modified variants 3rep-KTAAWN, 3rep-GVLEGV, and 3rep-ITVQQ, which were engineered to have reduced α-helix content, displayed varying degrees of β-sheet formation (7.5%, 22.6%, and 38.0%, respectively). Upon thermal induction at 45°C, 65°C, and 85°C, the secondary structure composition of all proteins underwent significant changes. Notably, after 65°C induction, 3rep-WT exhibited the highest β-sheet content of 41.5%. Meanwhile, 3rep-GVLEGV and 3rep-KTAAWN showed comparable β-sheet contents of 33.7% and 33.4% following induction at 65°C and 85°C, respectively. The most striking observation was that 3rep-ITVQQ achieved its highest β-sheet content of 81.3% after 65°C induction, nearly doubling that of 3rep-WT. This high β-sheet content is a critical factor contributing to the superior mechanical strength of spider silk fibers. The recombinant spidroin described herein exhibits a higher β-sheet content compared to the natural spidroin MaSp1, characterized by interlamellar cross-β-spine sheets.

In this study, we lyophilized three soluble recombinant spidroins and observed the surface morphology of the resulting samples in detail using scanning electron microscopy (SEM). The SEM images of 3rep-ITVQQ (a), 3rep-KTAAWN (b), and 3rep-GVLEGV (c) are shown in Supplementary Figure S3. These images allow us to analyze the microstructural features of these recombinant arachnid proteins in depth.

Under low magnification (Supplementary Figure S3a–i), we can see that the protein formed a more uniform film-like structure with a smooth surface and no obvious particles or holes. This indicates that 3rep-ITVQQ formed a more dense network structure during the lyophilization process. Under high magnification (Supplementary S3a–3ii, 3iii), it can be observed that the protein fibers have a more uniform diameter, and the fibers are intertwined with each other, forming a complex three-dimensional network. This structure may contribute to the mechanical strength and stability of the material.

For 3rep-KTAAWN, under low magnification (Supplementary Figure S3b–i), its surface morphology is similar to that of 3rep-ITVQQ, which also exhibits a film-like structure. However, under high magnification (Supplementary Figure S3b-ii, iii), we can observe that the diameter of the protein fibers varies more, and the interweaving between the fibers is not as tight as that of 3rep-ITVQQ. This may mean that 3rep-KTAAWN formed a looser network structure during the lyophilization process, which may affect its mechanical properties.

In contrast to the membranous structure of the former two, 3rep-GVLEGV showed an irregular block structure on its surface at low magnification (Supplementary Figure S3c–i). Under high magnification (Supplementary Figure S3c-ii, S4c-iii), aggregation of protein fibers into larger particles with distinct pores between the particles could be observed. This structural feature may be related to the self-assembly behavior of 3rep-GVLEGV, and the presence of pores may affect the density and mechanical properties of the material.

Combining the SEM image analyses of the three recombinant arachnid proteins, we can draw the following conclusions: in terms of structural densities, 3rep-ITVQQ has the densest network structure, which may have the best mechanical properties. 3rep-KTAAWN has a relatively loose structure, whereas 3rep-GVLEGV shows a porous block structure. In terms of fiber diameter consistency, 3rep-ITVQQ has more consistent fiber diameters, which may contribute to uniform mechanical properties. In contrast, the fiber diameter of 3rep-KTAAWN is more variable, which may affect the consistency of its properties. In terms of porosity and granularity, the porous and granular structure of 3rep-GVLEGV may reduce the density of the material but may also offer potential advantages for other applications, such as drug release.

### Self-assembly process verified by coarse-grained molecular dynamics

We applied the single-bead approximation to the MARTINI model (De Jong et al., 2013; Marrin et al., 2008; Periole et al., 2013) as an efficient way to calculate X-ray scattering from coarse-grained structures.

A snapshot is an analytical tool in molecular dynamics simulations that represents the exact state of a simulated system at a specific point in time. The recording of these states allows us to examine and analyze the dynamic behavior of the system in detail, including but not limited to the interactions between atoms, the trajectories of molecules, and the evolution of the overall structure of spidroin.

**FIGURE 4:**
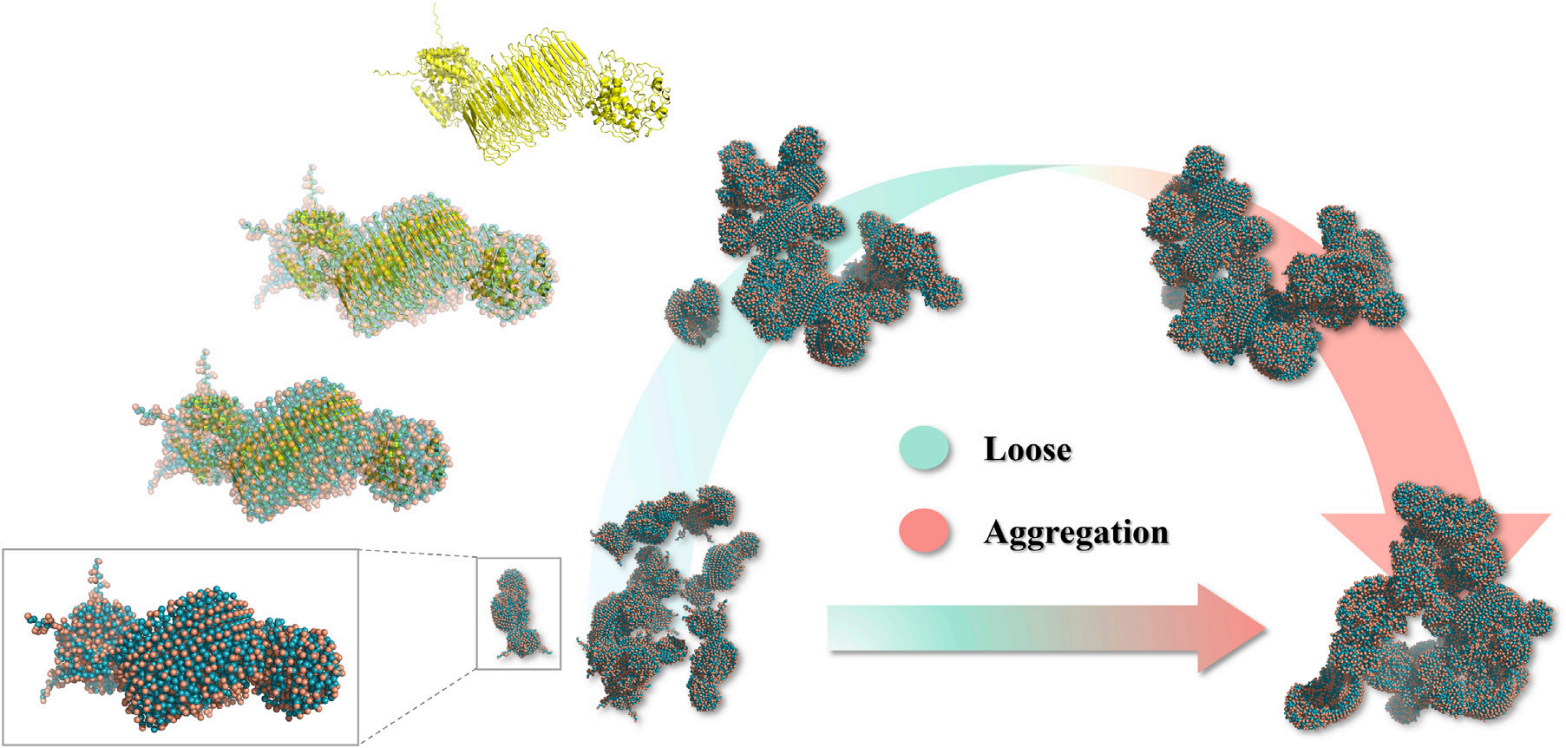
Snapshots of 24rep-ITVQQ at 0, 600, 800, and 1,000 ns.

The recombinant spidroin 24rep-ITVQQ in different moments in time conformations, as we can see, the monomers gradually gathered together over time, and it has shown a good self-assembly trend at 600 ns, which showed that the designed recombinant spidroin had good self-assembly ability (Figure 4). We also tested the distance between the two spidroin centers, and as the simulation time increased, the spidroin’s center distance decreased significantly, from a maximum of 25.665 ns to a minimum of 16.693 ns, which also proved that the two spidroin monomers slowly assembly together.

**FIGURE 5:**
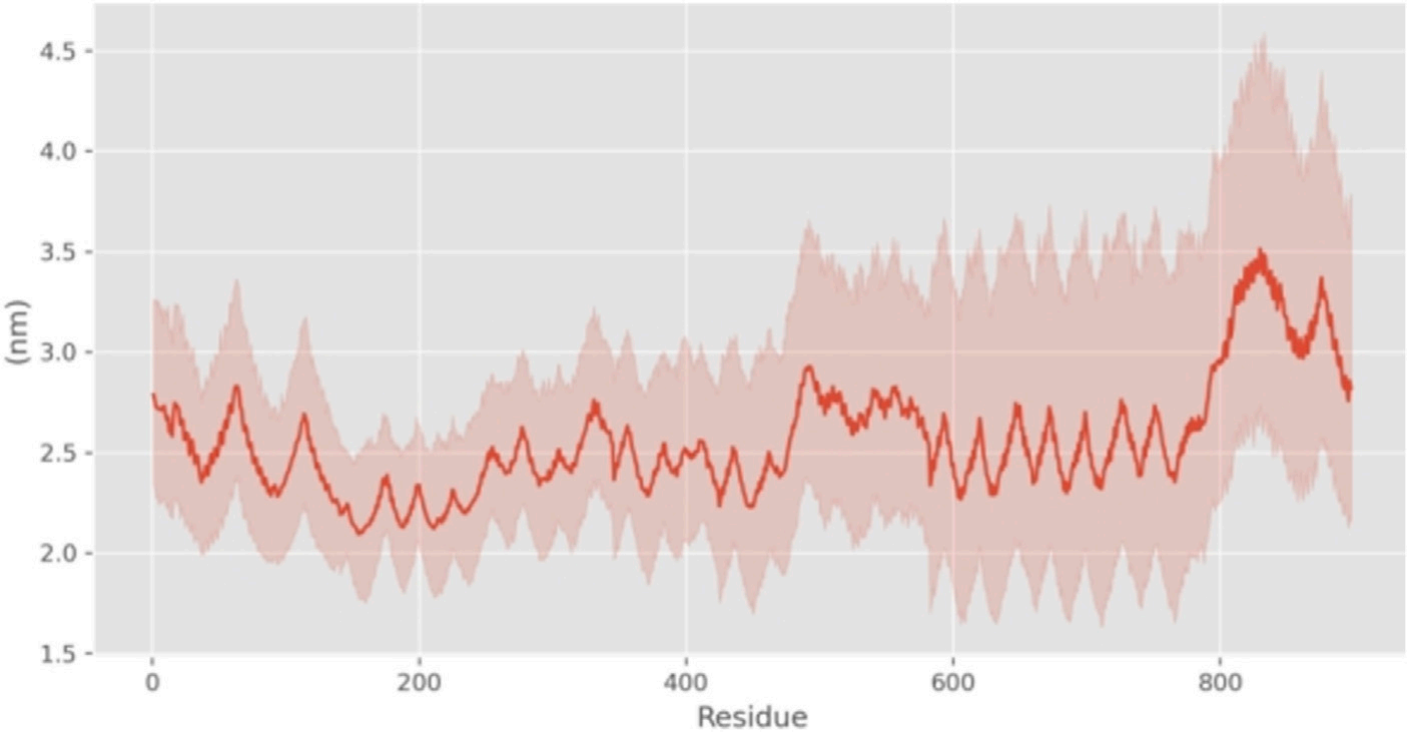
RMSF curves for different residues of 24rep-ITVQQ.

In addition, we calculated Root-Mean-Square Fluctuations (RMSF) to quantify the extent of deviation of individual atoms or residues from their average positions during the simulation. RMSF analysis focuses on local structural fluctuations. Typically, fluctuations are larger at the termini due to increased degrees of freedom. Significant fluctuations in the RMSF plot at positions other than the termini may indicate flexible regions within the molecule. Besides the substantial fluctuations observed for amino acid residues with high degrees of freedom at the N- and C-terminal ends, the RMSF values in the region of residues 50–70 (peaking at 4.5 Å) are notably higher compared to other regions, suggesting that this region might serve as a flexible, functional domain or a potential ligand-binding site for the protein (Figure 5).

**FIGURE 6:**
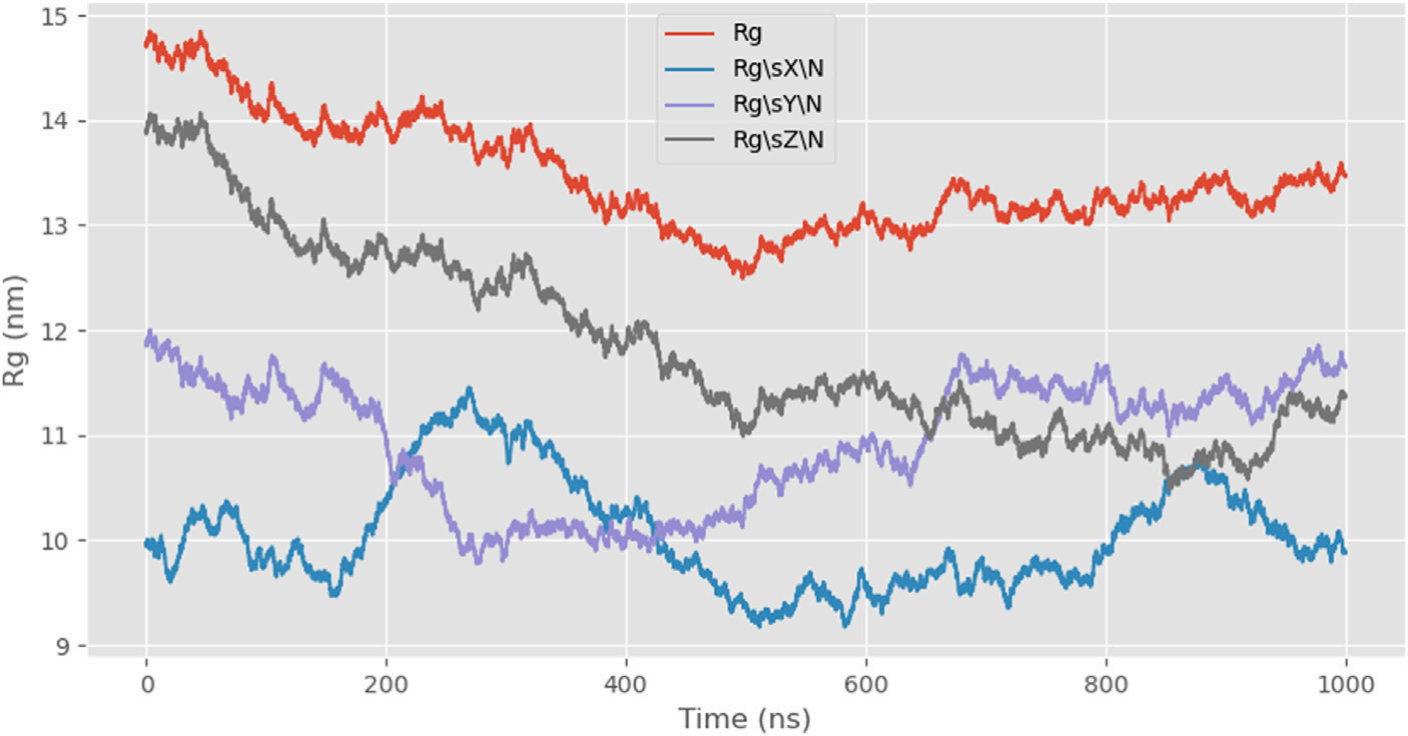
Rg curves of 24rep-ITVQQ over time.

Next, we simulated the radius of gyration (Rg) of the protein, which is a parameter describing the mass distribution relative to the center of mass. It indicates the average distance of all atoms from the center of mass and reflects the degree of molecular expansion or compaction. Rg can be used to assess whether the molecule undergoes significant conformational changes during the simulation and how its compactness evolves. The combination of snapshot analysis further supports the conclusion that 24rep-ITVQQ exhibits strong self-assembly properties, as evidenced by the Rg value decreasing from an initial 14.2 Å to 13.46 Å, indicating gradual folding and increased overall compactness (Figure 6).

We also validated the self-assembly performance of 24rep-ITVQQ by simulating the solvent-accessible surface area (SASA) and measuring the change in protein center-of-mass distance (COMD). SASA is a critical parameter in molecular dynamics simulations and structural biology, representing the surface area of a molecule accessible to solvent molecules. A larger SASA value typically indicates greater surface exposure and susceptibility to solvent interactions (e.g., water).

The protein center-of-mass distance (COMD) is defined as the straight-line distance between the centers of mass of two protein molecules (or different structural domains of the same protein). We measure this variation in molecular dynamics simulations primarily to track the dynamic behavior of the system, such as complex stability.

**FIGURE 7:**
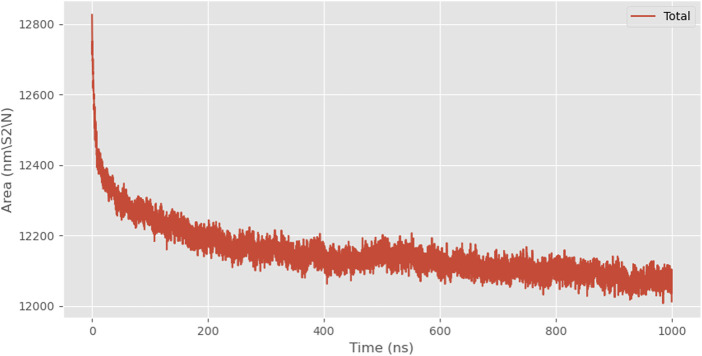
SASA analysis of 24rep-ITVQQ.

At the beginning of the simulation, the solvent-accessible surface area was approximately 12826.889 nm^2^, and by the end of the simulation, it decreased to about 12012.084 nm^2^, representing a reduction of approximately 6.4% (Figure 7).

**FIGURE 8:**
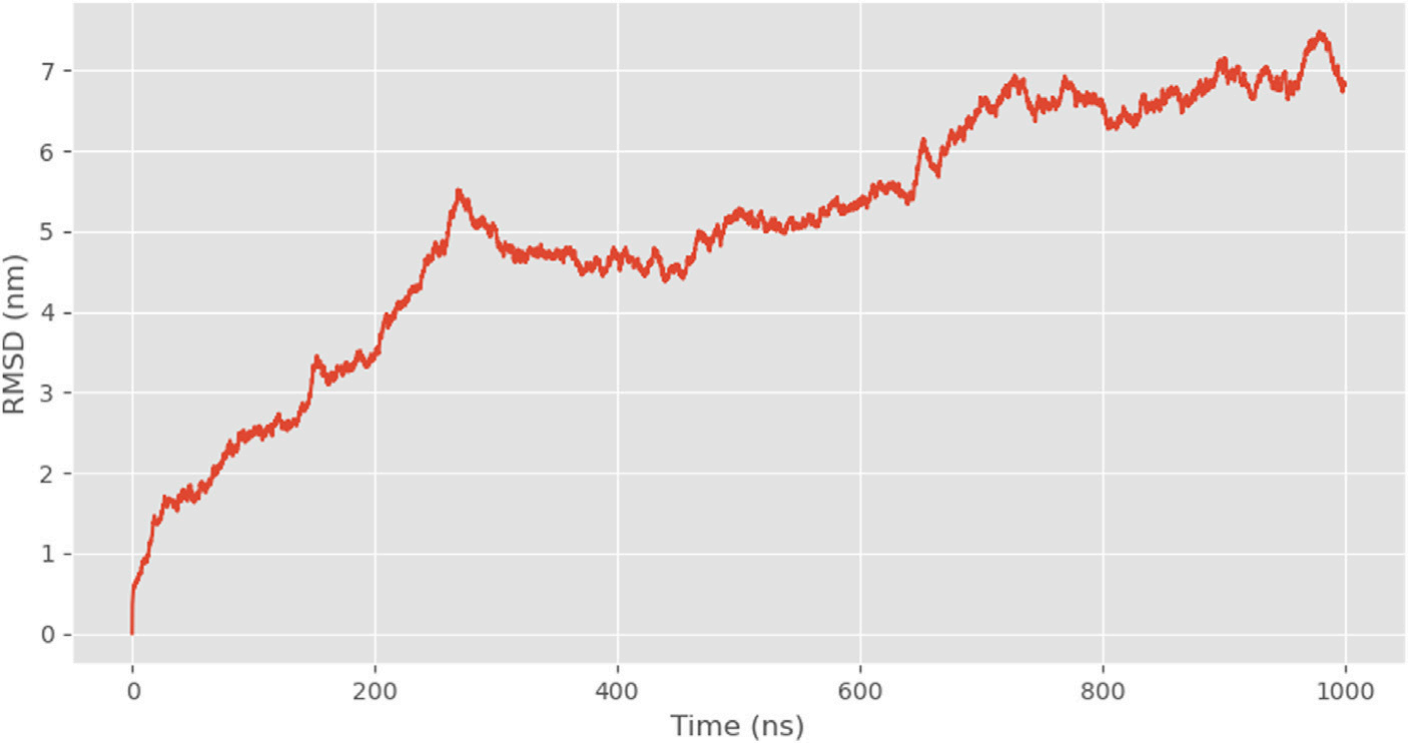
Protein center-of-mass distance variation curves for 24rep-ITVQQ.

As the simulation progressed, the solvent-accessible surface area of the protein gradually decreased, indicating protein aggregation. Combined with the trend of compacting protein conformation observed in snapshots, it was hypothesized that the decrease in SASA was mainly caused by structural folding rather than mere aggregation. However, the COMD change curve shows that the distance between the 24rep-ITVQQ polypeptide chains shortened in the late stages of the simulation (from an initial 25 Å to 18 Å), suggesting possible weak interaction-driven local aggregation. A further distinction between folding and aggregation effects would require all-atom simulations or experimental validation (Figure 8).

### Analysis of the self-assembly capability of 24rep-ITVQQ protein

**FIGURE 9:**
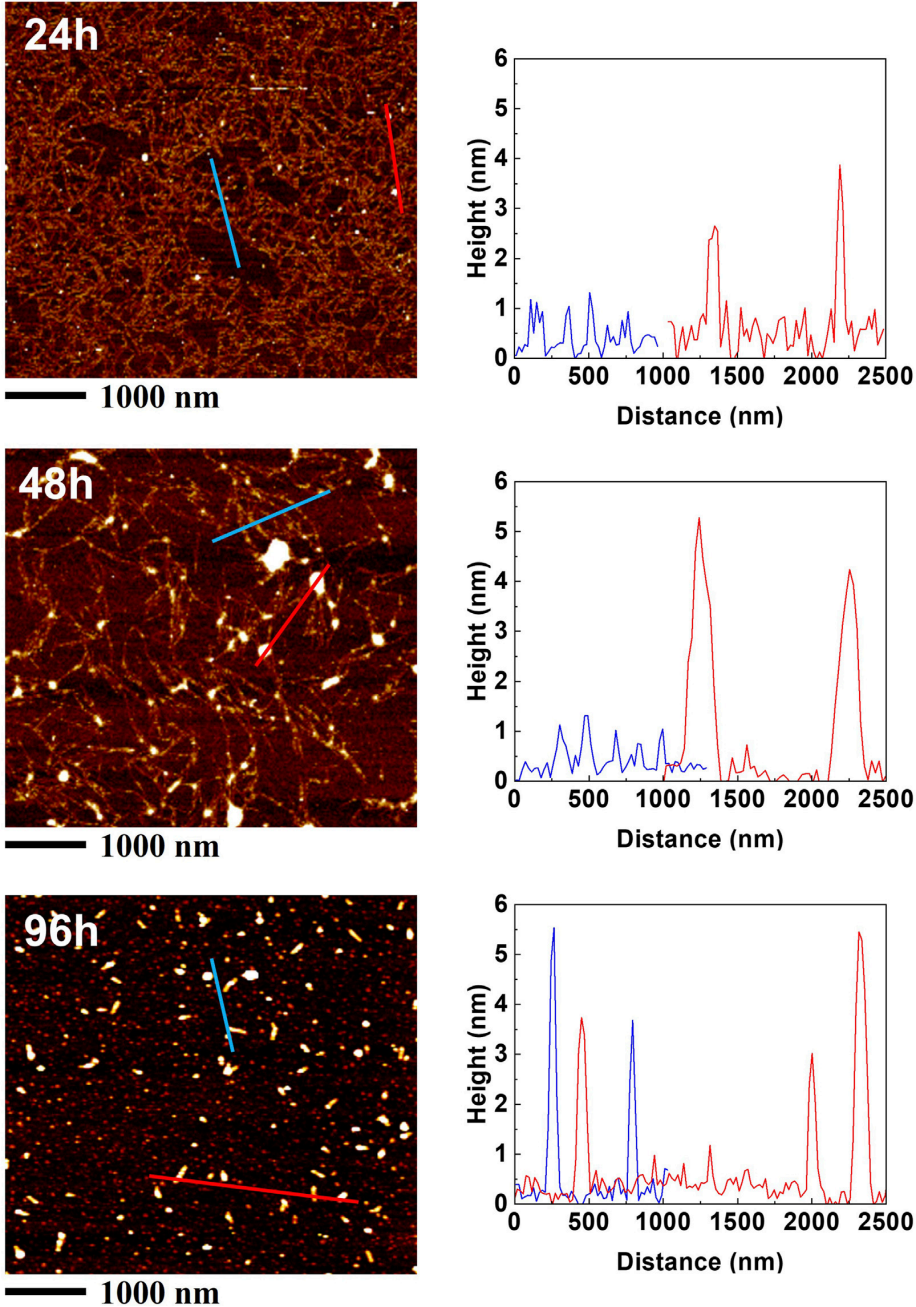
The self-assembly process of 24rep-ITVQQ.

The characterization of the self-assembly process of the 24rep-ITVQQ protein using AFM reveals that at the initial time point, the 24rep-ITVQQ protein exhibits two distinct morphologies. Specifically, a network structure is formed by multiple molecular chains interconnecting and stacking together, with a diameter of approximately 0.5–1 nm (Figure 9 red line). Subsequently, on this molecular chain network foundation, proteins further aggregate to form nanoparticles with diameters around 3 nm (Figure 9 blue line). As assembly progresses over time, proteins continue to coalesce; after 48 h, both morphologies remain observable. However, there is a notable increase in nanoparticle quantity and their diameters have expanded to between 4 and 6 nm. Ultimately, following a self-assembly period of 96 h, most proteins cluster together into short rod-like nanofibers.

This observation further corroborates our CGMD simulation results for the 24rep-ITVQQ protein and provides significant experimental evidence for subsequent studies on the directed assembly mechanisms of silk fibroin biomimetic materials.

### Morphology and characterization of 24rep-ITVQQ electrospun nanofibers

**FIGURE 10:**
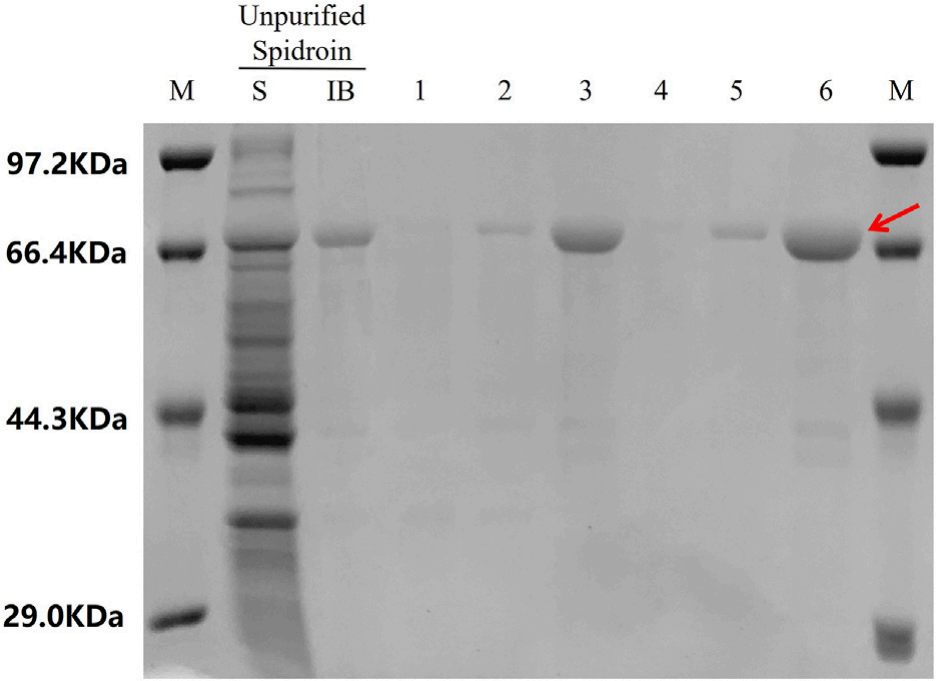
Purification of 24rep-ITVQQ. LaneKey: S stands for soluble fractions; IB stands for inclusion bodies. Lane 1: S after low-speed centrifugation after the first wash. Lane 2: S after high speed centrifugation after the first wash. Lane 3: IB after the first centrifugation. Lane 4: S after low speed centrifugation in the second wash. Lane 5: S after high speed centrifugation in the second wash. Lane 6: 2 × IB after the second centrifugation.

**FIGURE 11:**
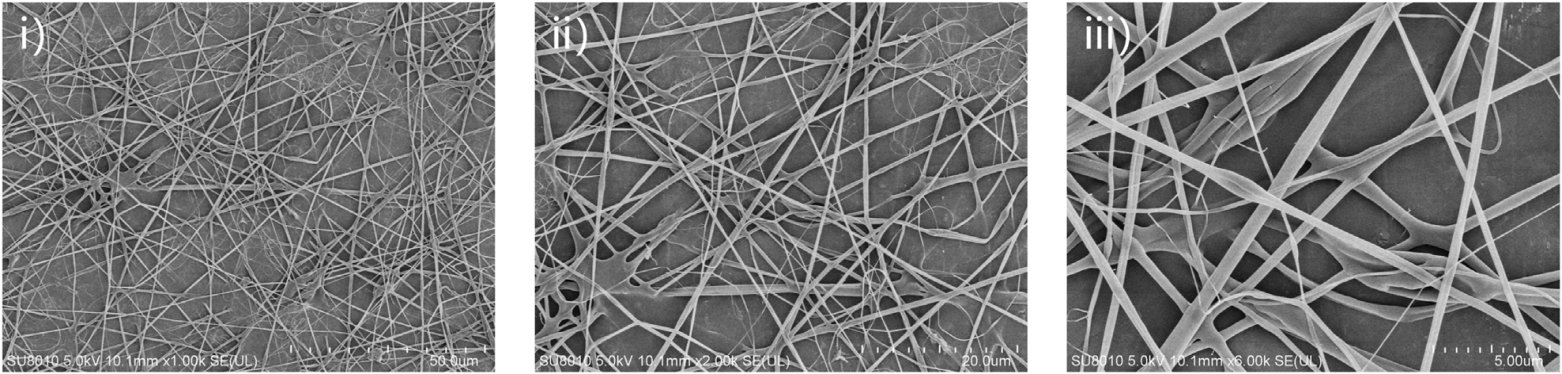
SEM micrographs of the 24rep-ITVQQ electrospun nanofibers. Scale bars represent 50 μm (i), 20 μm (ii), and 5 μm (iii) respectively.

The purity of 24rep-ITVQQ (Figure 10) ensured consistent electrospinning performance. The SEM morphology of the electrospun fibers derived from the recombinant spidroin 24rep-ITVQQ is illustrated in Figure 11. The fibers produced from 24rep-ITVQQ demonstrate a dense arrangement with a uniform distribution, free from any spherical or droplet-like agglomerations. This observation suggests enhanced interfacial interactions and, at higher magnification (x6.0 W), reveals a branched fiber network characterized by localized interconnections. Such features may be attributed to the hydrophilic ITVQQ motif, which promotes solvent accessibility and dynamic chain reorganization, exhibiting remarkable self-assembly properties.

Moreover, ultra-fine fibers measuring less than 10 nm are also present within the 24rep-ITVQQ matrix, indicating its potential for biomedical applications that require nanoscale porosity, such as drug delivery systems or tissue engineering scaffolds. The significant increase in crosslinking points and improved continuity of the fiber network can be ascribed to enhanced cooperative interactions among molecular chains facilitated by high repeat structural domains.

## Discussion

This study optimized prokaryotic expression by designing water-soluble, β-sheet-rich spidroin mimics, and in this work, spidroins achieved significant improvements in yield and solubility compared to conventional systems. Below, we present these findings in the broader context of spider silk research, while highlighting the advancements and limitations of our approach.

We first used deep learning algorithms to intelligently screen and semi-rationally design the β-sheet structure of the microcrystalline region of spidroins. Based on the statistical learning theory, the β-sheet feature motif information from different biological sources was collected from the NCBI/PDB database, and the features were further extracted and classified to construct the β-sheet feature sequence database. By analyzing the sequence information in the protein database, we predicted and screened the arachnid sequences with potential self-assembly ability. Using the modified MaSp1 as a template, replacing the polyalanine sequences in the repeat region and performing codon optimization, we designed a class of folded sheet interlamellar microcrystalline region motifs capable of forming a cross-β-spine feature, which provides an effective template for the subsequent substitution of key feature motifs of the recombinant spidroins and lays the foundation for their modification. This strategy addresses the challenges of expressing high molecular weight, repetitive, and GC-rich spidroins in prokaryotic systems, which typically result in low yields, premature transcription termination, and insoluble inclusion bodies (Rising et al., 2005). Our modified recombinant spidroins achieved a soluble yield of 0.99 g/L in *E. coli*, a significant improvement over the unmodified MaSp1 system, which typically yields less than 0.5 g/L under comparable conditions (Yesilyurt et al., 2015). The application of deep learning techniques, such as AlphaFold3 for protein structure prediction, has paved new avenues for designing β-sheet motifs in recombinant spidroins. This innovation offers promising pathways for developing biomaterials with exceptional mechanical properties. The strategies for synthesizing recombinant spidroins through genetic engineering, along with multiscale simulations to aid in material design, have been thoroughly reviewed (Zheng and Ling, 2019). Researchers have developed a modular functionalization strategy for recombinant spider silk using SpyTag/SpyCatcher chemistry and AlphaFold2-aided design, allowing for the creation of multifunctional silk materials (Jiang et al., 2023). Additionally, a generative large-language model has been proposed for designing spidroin sequences with tailored mechanical properties, expanding the silkome dataset and facilitating sequence-structure analyses (Lu et al., 2024). Such methodologies are equally applicable to other repetitive protein systems, including collagen, further expanding their potential applications in the biomedical field.

Subsequently, employing the coarse-grained molecular dynamics simulation (CGMD) technique, we utilized the MARTINI model to simulate the self-assembly behavior of the designed recombinant arachnid proteins. This approach approximates complex protein structures using a single bead representation, providing an efficient method for calculating X-ray scattering of coarse-grained structures. The types of beads used for these calculations and their elemental compositions are detailed in Supplementary Table S1. This process simplifies intricate protein molecules into computationally manageable models, enabling us to observe their behavior in a simulated environment and gain insights into their structural and dynamic properties. A snapshot analysis of 24rep-ITVQQ was conducted initially (Figure 4), revealing that the engineered recombinant spidroins exhibited superior self-assembly characteristics. Additionally, Root Mean Square Deviation (RMSD) analysis was performed to quantify structural changes over time, specifically the conformational deviations relative to the initial state. The RMSD values were plotted as a function of simulation time. Such graphs provide a visual representation of the structural fluctuations of the molecule during the simulation. We measured and showed the RMSD curve of 24rep-ITVQQ as a function of time. From the curves, it can be seen that at the end of the simulation (1000 ns) the RMSD value is 6.82 (Supplementary Figure S2), which indicates that the system has reached equilibrium or a steady state. Both the RMSD and RMSF analyses reveal structural stability and dynamic fluctuations of proteins during simulations, whereas the Rg and SASA analyses provide information on protein important information on structural compactness and solvent interactions (Figure 6). The Rg was shown to decrease from the initial 14.2 Å to 13.46 Å (ΔRg = 0.74 Å), suggesting that the protein underwent conformational reorganization during the simulation process and gradually shifted to a more compact folded state. To further investigate whether the recombinant protein undergoes aggregation, we conducted simulations of the SASA to monitor changes in the center-of-mass distance. Initially, the SASA was 12826.889 square nanometers, decreasing to 12012.084 square nanometers by the end of the simulation. The progressive reduction in SASA suggests that protein aggregation is occurring. Similarly, the COM distance decreased from 24.95 nm at the start of the simulation (0 ns) to 18.887 nm at its conclusion (1,000 ns), indicating partial aggregation within the protein (Figures 7, 8). These findings provide additional evidence supporting the superior self-assembly properties of 24rep-ITVQQ. The results of CGMD simulations using the MARTINI force field demonstrate that the engineered recombinant spidroin 24rep-ITVQQ exhibits excellent self-assembly performance, offering new insights into the self-assembly mechanisms of recombinant spidroins. This study also systematically investigated the expression optimization and purification of recombinant spidroins and the characterization of their structural properties. Taking recombinant spidroin 6rep-ITVQQ as an example, to further improve the properties of recombinant spidroins and construct polyploids of the rest of screened recombinant spidroins by enzymatic ligation. By adjusting the IPTG concentration, induction temperature, and duration, the modified spidroins (3rep-GVLEGV as an example) we significantly increased the yield and solubility of the target protein from 0.047 g/L to 0.99 g/L, an increase of about 0.943 g/L, which is about 21.06 times higher.

In terms of protein purification, for soluble proteins, we added His tags at the beginning of the construction and used nickel column affinity purification technology to obtain electrophoretically pure proteins and verified by SDS-PAGE, and the purity of recombinant spidroins was up to 95%. The concentration of 3rep recombinant spidroins reached 480 mg/L (3rep-ITVQQ), 510 mg/L (3rep-KTAAWN), 510 mg/L (3rep-ITVQQ), and 990 mg/L (3rep-GVLEGV) (Figure 2B). 6rep recombinant arachnid protein concentrations reached 631 mg/L (6rep-KTAAWN) and 506 mg/L (6rep-ITVQQ) (Supplementary Figure S4). After quantification of protein concentration by greyscale scanning, among them, 6rep-GVLEGV was lower in protein expression compared to 6rep-ITVQQ and 6rep-KTAAWN, so its purification operation was not performed.

SEM showed that different recombinant spidroins exhibited different microstructures (Supplementary Figure S3). For example, 3rep-ITVQQ formed a denser network structure, while 3rep-GVLEGV showed a porous block structure. By electrostatic spinning, we successfully prepared 24rep-ITVQQ nanofibers (Figure 11). Notably, SEM results showed that the mutant 3rep-ITVQQ β-sheet content was close to the level observed in native silk fibers, with a significant increase in β-sheet compared to 3rep-WT, which also highlighted the success of the sequence modification we screened for, as confirmed by circular dichroism and structural simulations. At room temperature, the engineered variants (3rep-KTAAWN, 3rep-GVLEGV, and 3rep-ITVQQ) all already showed high β-sheet content (7.5%–38.0%), which contrasts with the 3rep-WT. We further amplified this trend by heat induction: the β-sheet content of 3rep-WT was 41.5% at 65 °C, whereas the 3rep-ITVQQ variant demonstrated an unprecedented β-sheet content of 81.3% under identical conditions, representing a nearly twofold increase compared to the 3rep-WT (41.5%). The β-sheet nanocrystals in the amorphous zone of the spider silk hierarchical structure, as studied by Nova et al. give it superior mechanical properties as well (Nova et al., 2010; Verma et al., 2022). We keep mentioning the importance of β-sheet, and this extraordinary structural transformation is consistent with the superb mechanical strength expected in synthetic fibers since the interlayer structure of folded sheets characterized by cross-β-spine is crucial for the load-bearing capacity.

In comparison with existing methods, although our method shows a significant improvement in expression efficiency and solubility, it must be recognized that prokaryotic systems have limitations in replicating post-translational modifications and biocompatibility of natural spiders. Eukaryotic expression systems, such as yeast and mammalian cells, have been shown to produce more biocompatible spider silk, making them more suitable for biomedical applications (Widmaier et al., 2009). In addition, our studies have focused mainly on structure- and expression-related results, leaving the mechanical properties of engineered fibers largely unexplored. Although our CGMD simulations provided insights into the self-assembly process, further experimental validation is needed to assess the durability and functionality of these materials for practical applications such as tissue engineering and drug delivery (Numata and Kaplan, 2010). In contrast, other studies have provided detailed mechanical properties, such as tensile strength and elasticity, which are essential for evaluating the performance of recombinant spider silks in practical applications (Lazaris et al., 2002). In the future, we will focus on exploring the practical applications of the designed spidroins.

In conclusion, this study is an important step forward in the production of spidroins by using computer design and bioengineering to overcome traditional barriers to protein expression. The findings provide a promising blueprint for the large-scale production of high-performance biomimetic fibers. However, future work should focus on mechanical testing, biocompatibility, and long-term stability to realize the full potential of these materials in advanced applications.

## Data Availability

The datasets presented in this study can be found in online repositories. The names of the repository/repositories and accession number(s) can be found in the article/Supplementary Material.
