# Supplementary material for: High-yield spidroin mimics for bioinspired fibers via computational design
Source: Front Bioeng Biotechnol. 2025 Apr 24;13:1587546. doi: 10.3389/fbioe.2025.1587546 (PMC12058734; doi:10.3389/fbioe.2025.1587546)
Supplement: Supplementary file 1 [file DataSheet1.docx]

***Supplementary Material***

- 1. **Supplementary Tables**

**Supplementary Table 1.** Bead types used for the coarse-grained X-ray scattering calculations and their elemental formulae.

| Number of atoms | | | | | | | | | | | | | | | |
| --- | --- | --- | --- | --- | --- | --- | --- | --- | --- | --- | --- | --- | --- | --- | --- |
| AA | Bead | C | H | N | O | S | f(q=0) | AA | Bead | C | H | N | O | S | f(q=0) |
| ALA | BB | 3 | 5 | 1 | 1 | 0 | 9.04 | MET | BB | 2 | 2 | 1 | 1 | 0 | 10.69 |
| ALA | BB | 3 | 5 | 1 | 1 | 0 | 10.69 | MET | SC1 | 3 | 7 | 0 | 0 | 1 | 5.86 |
| ARG | SC1 | 3 | 6 | 0 | 0 | 0 | -2.79 | PHE | BB | 2 | 2 | 1 | 1 | 0 | 10.69 |
| ARG | SC2 | 1 | 5 | 3 | 0 | 0 | 15.39 | PHE | SC1 | 3 | 3 | 0 | 0 | 0 | -0.63 |
| ASN | BB | 2 | 2 | 1 | 1 | 0 | 10.69 | PHE | SC2 | 2 | 2 | 0 | 0 | 0 | -0.42 |
| ASN | SC1 | 2 | 4 | 1 | 1 | 0 | 9.25 | PHE | SC3 | 2 | 2 | 0 | 0 | 0 | -0.42 |
| ASP | BB | 2 | 2 | 1 | 1 | 0 | 10.69 | PRO | BB | 2 | 1 | 1 | 1 | 0 | 11.41 |
| ASP | SC1 | 2 | 2 | 0 | 2 | 0 | 9.48 | PRO | SC1 | 3 | 6 | 0 | 0 | 0 | -2.79 |
| GLN | BB | 2 | 2 | 1 | 1 | 0 | 10.69 | SER | BB | 2 | 2 | 1 | 1 | 0 | 10.69 |
| GLN | SC1 | 3 | 6 | 1 | 1 | 0 | 8.32 | SER | SC1 | 1 | 3 | 0 | 1 | 0 | 3.3 |
| GLU | BB | 2 | 2 | 1 | 1 | 0 | 10.69 | THR | BB | 2 | 2 | 1 | 1 | 0 | 10.69 |
| GLU | SC1 | 3 | 4 | 0 | 2 | 0 | 8.55 | THR | SC1 | 2 | 5 | 0 | 1 | 0 | 2.37 |
| GLY | BB | 2 | 3 | 1 | 1 | 0 | 9.97 | TRP | BB | 2 | 2 | 1 | 1 | 0 | 10.69 |
| HIS | BB | 2 | 2 | 1 | 1 | 0 | 10.69 | TRP | SC1 | 3 | 2 | 0 | 0 | 0 | 0.09 |
| HIS | SC1 | 2 | 2 | 0 | 0 | 0 | -0.42 | TYP | SC2 | 2 | 2 | 1 | 0 | 0 | 5.74 |
| HIS | SC2 | 1 | 1 | 1 | 0 | 0 | 5.95 | TYP | SC3 | 2 | 2 | 0 | 0 | 0 | -0.42 |
| HIS | SC3 | 1 | 1 | 1 | 0 | 0 | 5.95 | TYP | SC4 | 2 | 2 | 0 | 0 | 0 | -0.42 |
| ILE | BB | 2 | 2 | 1 | 1 | 0 | 10.69 | TYR | BB | 2 | 2 | 1 | 1 | 0 | 10.69 |
| ILE | SC1 | 4 | 9 | 0 | 0 | 0 | -4.44 | TYR | SC1 | 3 | 3 | 0 | 0 | 0 | -0.63 |
| LEU | BB | 2 | 2 | 1 | 1 | 0 | 10.69 | TYR | SC2 | 2 | 2 | 0 | 0 | 0 | -0.42 |
| LEU | SC1 | 4 | 9 | 0 | 0 | 0 | -4.44 | TYR | SC3 | 2 | 2 | 0 | 1 | 0 | 4.53 |
| LYS | BB | 2 | 2 | 1 | 1 | 0 | 10.69 | VAL | BB | 2 | 2 | 1 | 1 | 0 | 10.69 |
| LYS | SC1 | 3 | 6 | 0 | 0 | 0 | -2.79 | VAL | SC1 | 3 | 7 | 0 | 0 | 0 | -3.51 |
| LYS | SC2 | 1 | 5 | 1 | 0 | 0 | 3.07 |  |  |  |  |  |  |  |  |

- 1. **Supplementary Figures**


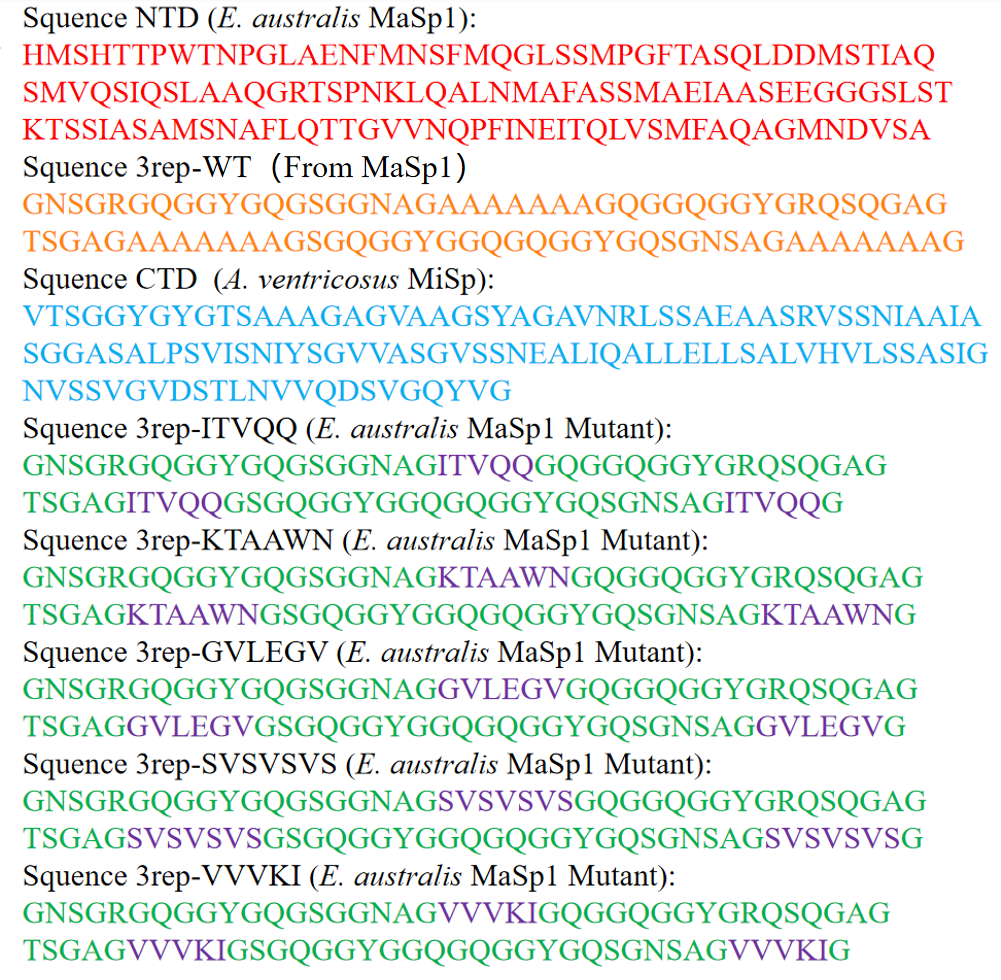


**Supplementary Figure 1.** Amino acid sequences of NT, CT, 3rep-WT and modified recombinant spidroins.


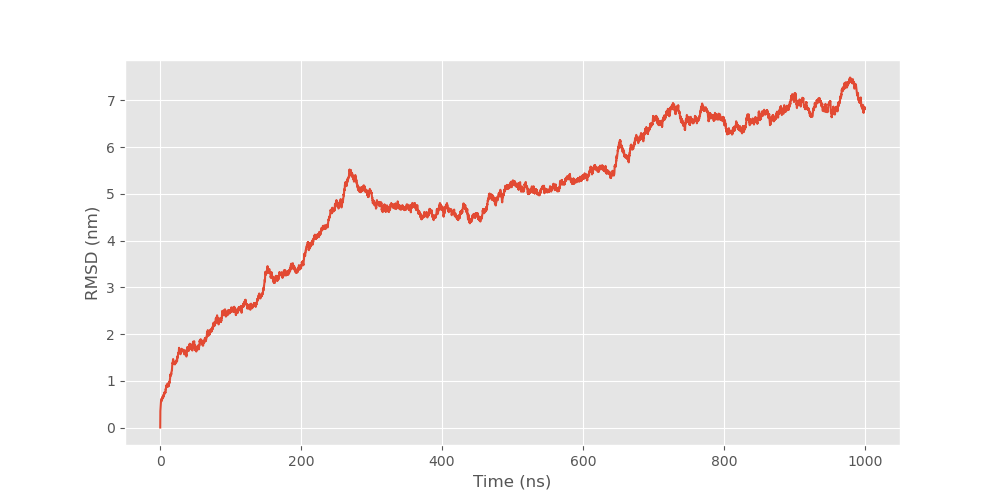


**Supplementary Figure 2.** RMSD curves of 12rep-ITVQQ over time.

**a**


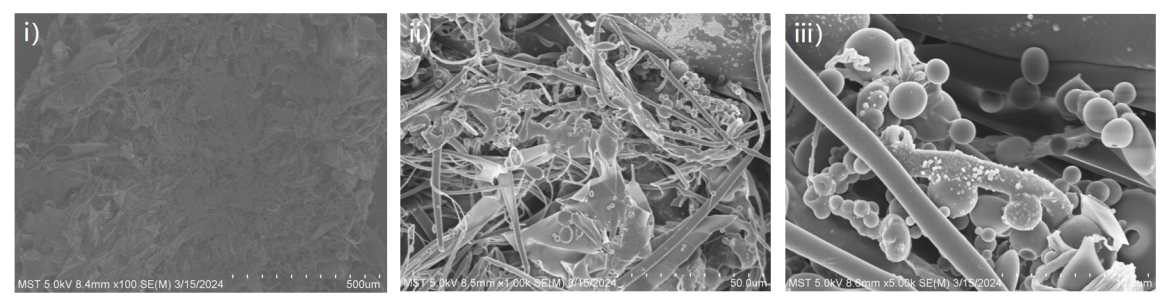

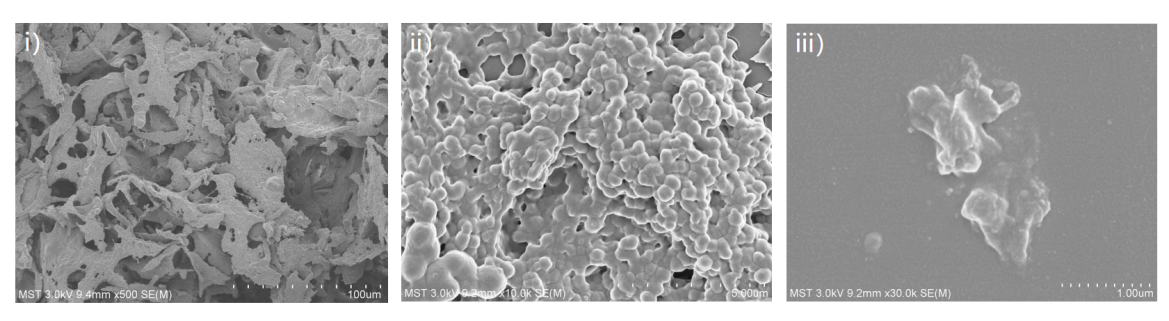

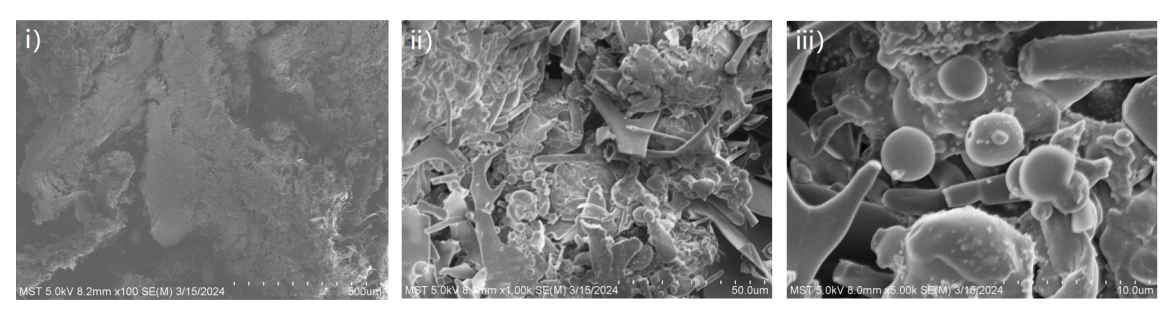


**c**

**b**

**Supplementary Figure 3.** SEM images of 3rep-ITVQQ **(a)**, 3rep-KTAAWN **(b)**, and 3rep-GVLEGV **(c)**.

Scale bars represent 500 μm (i), 50 μm (ii), and 10 μm (iii) respectively. The images depict the surface morphology of the proteins, with (a) showing a relatively smooth and dense network structure for 3rep-ITVQQ, (b) presenting a less compact structure for 3rep-KTAAWN, and (c) illustrating an irregular blocky structure with visible pores for 3rep-GVLEGV."

**b**

**a**


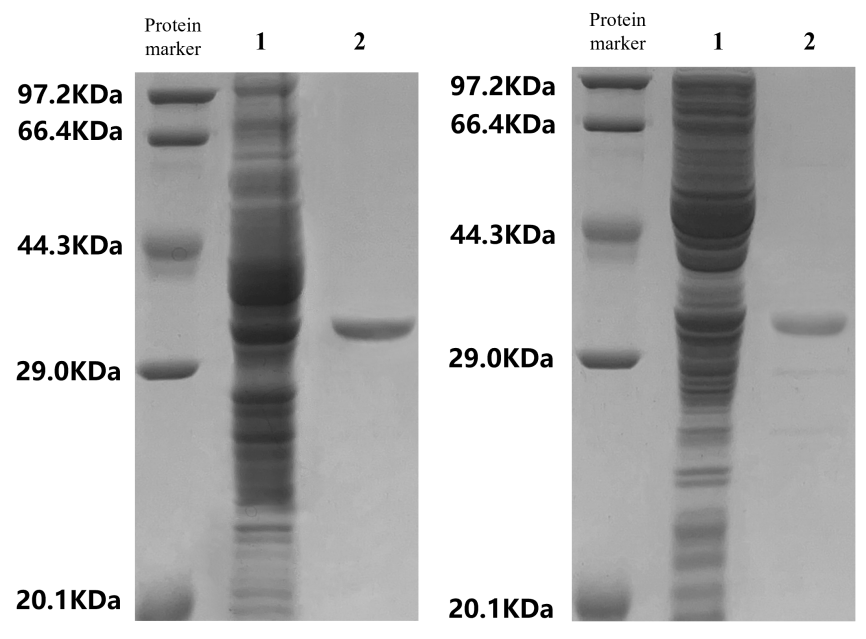


**Supplementary Figure 4.** Purification of 6rep-KTAAWN **(a)** and 6rep-ITVQQ **(b)** .

LaneKey: Lane 1: the concentrated cell lysis of spidroin; Lane 2: the purified spidroin.
